# Lower health-related quality of life predicts all-cause hospitalization among HIV-infected individuals

**DOI:** 10.1186/s12955-018-0931-x

**Published:** 2018-05-30

**Authors:** Leonard Emuren, Seth Welles, Marcia Polansky, Alison A. Evans, Grace Macalino, Brian K. Agan, Infectious Disease Clinical Research Program HIV Working Group, S. Chambers, M. Fairchok, A. Kunz, C. Schofield, J. Powers, E. Tramont, S. Banks, K. Kronmann, T. Lalani, K. St. Clair, R. Tant, M. Bavaro, R. Deiss, A. Diem, N. Kirkland, R. Maves, S. Merritt, T. O’Bryan, J. Okulicz, C. Rhodes, J. Wessely, T. Ferguson, J. Hawley-Molloy, B. Agan, M. Byrne, X. Chu, M. Glancey, G. Macalino, E. Parmelee, J. Pavlin, X. Wang, S. Won, P. Wright, S. Peel, J. Blaylock, H. Burris, C. Decker, A. Ganesan, R. Ressner, D. Wallace, T. Whitman

**Affiliations:** 1 Department of Epidemiology and Biostatistics, Dornsife School of Public Health, Drexel University, Philadelphia, PA USA; 2 Department of Pediatrics, Eastern Virginia Medical School, Norfolk, VA USA; 3 Children’s Hospital of the King’s Daughters, 601 Children’s Lane, Norfolk, VA 23507 USA; 4 Infectious Disease Clinical Research Program, Department of Preventive Medicine and Biostatistics, Uniformed Services University of the Health Sciences, Bethesda, MD USA; 5 Henry M. Jackson Foundation for the Advancement of Military Medicine, Inc., Bethesda, MD USA

**Keywords:** HIV, Human immunodeficiency virus, HRQOL, Health-related quality of life, HAART, Highly active antiretroviral therapy, PCSS, Physical component summary scores, MCSS, Mental component summary scores, Hospitalization

## Abstract

**Background:**

Health-related quality of life (HRQOL) is a patient-centered outcome measure used in assessing the individual’s overall functional health status but studies looking at HRQOL as a predictive tool are few. This work examines whether summary scores of HRQOL are predictive of all-cause hospitalization in the US Military HIV Natural History Study (NHS) cohort.

**Methods:**

The Short Form 36 (SF-36) was administered between 2006 and 2010 to 1711 NHS cohort members whose hospitalization records we had also obtained. Physical component summary scores (PCSS) and mental component summary scores (MCSS) were computed based on standard algorithms. Terciles of PCSS and MCSS were generated with the upper terciles (higher HRQOL) as referent groups. Proportional hazards multivariate regression models were used to estimate the hazard of hospitalization for PCSS and MCSS separately (models 1 and 2, respectively) and combined (model 3).

**Results:**

The hazard ratios (HR) of hospitalization were respectively 2.12 times (95% CI: 1.59–2.84) and 1.59 times (95% CI: 1.19–2.14) higher for the lower and middle terciles compared to the upper PCSS tercile. The HR of hospitalization was 1.33 times (95% CI: 1.02–1.73) higher for the lower compared to the upper MCSS tercile. Other predictors of hospitalization were CD4 count < 200 cells/mm^3^ (HR = 2.84, 95% CI: 1.96, 4.12), CD4 count 200–349 cells/mm^3^ (HR = 1.67, 95% CI: 1.24, 2.26), CD4 count 350–499 cells/mm^3^ (HR = 1.41, 95% CI: 1.09, 1.83), plasma viral load > 50 copies/mL (HR = 1.82, 95% CI: 1.46, 2.26), and yearly increment in duration of HIV infection (HR = 0.94, 95% CI: 0.93, 0.96) (model 3).

**Conclusion:**

After controlling for factors associated with hospitalization among those with HIV, both PCSS and MCSS were predictive of all-cause hospitalization in the NHS cohort. HRQOL assessment using the SF-36 may be useful in stratifying hospitalization risk among HIV-infected populations.

## Background

Health-related quality of life (HRQOL) is primarily used as a patient-centered outcome measure to assess the individual’s overall functional health status and for evaluating therapeutic interventions in chronic diseases including human immunodeficiency virus (HIV) infection and acquired immune deficiency syndrome (AIDS) [1, 2]. However, a few studies have also utilized HRQOL as a prognostic tool for predicting survival in people living with HIV/AIDS (PLWHA) [3–6], showing that HRQOL is useful as a risk stratification tool in HIV-infected individuals both in clinical trials and observational studies.

While HIV remains incurable, successful treatment with highly active antiretroviral therapy (HAART) has resulted in prolonged survival among PLWHA [7–9] and with the steady incidence of HIV in the United States [9], the prevalence of the disease and, by extension, the burden of the disease on the healthcare system will continue to rise. To mitigate the increasing burden of the disease on the healthcare system and improve the quality of life of infected individuals, it is important that PLWHA are clinically stable and in optimal functional health, free from medical/mental comorbidities or opportunistic infections, and have minimal hospitalizations. Poor HRQOL measures have been associated with higher utilization of healthcare resources among patients with other chronic diseases [10–12]. Among HIV-infected individuals, HRQOL has been shown to be associated with hospitalization and emergency department utilization [5]. The rate of hospitalization in the U.S. Military HIV Natural History Study (NHS) cohort was previously reported to be as high as 137 per 1000 person years (PYs) [13]. Given this high rate of hospitalization, it is important to evaluate factors that may predict hospitalization such as HRQOL, in the hope that appropriate interventions directed at modifiable risk factors such as CD4 count and medical/mental comorbidities that impact HRQOL can be instituted with the ultimate goal of reducing the high hospitalization rate.

While HRQOL may be measured using various instruments, the Research and Development (RAND) Short Form 36 (SF-36) is one of the more commonly used instruments both in clinical trials and observational studies. Although the Medical Outcome Studies for HIV questionnaire has been used in previous studies to predict mortality [4, 6], this instrument is disease-specific and its measured health dimensions are slightly different from that of the SF-36. Furthermore, an instrument’s ability in predicting mortality may not necessarily prove its usefulness in predicting other relevant clinical end-points such as hospitalization. To the best of our knowledge there has been only one study that has specifically looked at how HRQOL predicts hospitalization in HIV-infected individuals, but this study used a different instrument, the EuroQol [5], and adjusted for only CD4 count and HIV plasma viral load. In this research, we investigate the usefulness of the RAND SF-36 in predicting hospitalization in the NHS cohort. Because HRQOL reflects an individual’s overall physical and mental functional health status, we hypothesize that participants with lower HRQOL are more likely to be hospitalized compared to participants with higher HRQOL over the period of follow-up.

## Methods

### Study cohort

The NHS is a prospective multicenter continuous enrollment observational cohort of HIV-infected active duty military personnel and other beneficiaries from the Army, Navy/Marines and Air Force enrolled since 1986 [14–17]. Participants are followed at six medical centers in the U.S. Demographic data are collected at baseline and updated while medical and medication histories and standard laboratory studies are collected biannually. Blood samples obtained from participants in this cohort from scheduled visits are stored in a repository. All NHS participants provided informed consent, and approval for this research was obtained from the institutional review board at each participating site.

### Study participants

The SF-36 questionnaire was administered to NHS participants at every other study visit (approximately 12-month intervals) from April 2006 to September 2010. For these analyses, one SF-36 response per calendar year was captured, using the last measurement if more than one survey was completed in a calendar year. Baseline was defined as the earliest measure meeting these criteria. We used the CD4 count and viral load values closest in time to the HRQOL measure, usually the same visit.

### Definitions and variable selections

#### Hospitalization

Participants’ dates of hospitalization, diagnoses at hospitalization, and number of days of hospitalization were retrieved from their medical records and through coordinator interviews. Furthermore, the military healthcare system operates a centralized electronic health records system that enables investigators to track participants’ records and hospitalizations. Hospitalization was the outcome of interest, and we considered only the first admission of participants between April 2006 and September 2010 for the purposes of this study. To establish a temporal relationship, we ensured that date of completed questionnaire preceded the date of hospitalization. Hospitalization was coded as ‘yes’ if participant was hospitalized after the first completed SF-36 questionnaire and ‘no’ if participant was not hospitalized after the first completed questionnaire for the duration of follow-up. Some common causes of hospitalization include bacterial, viral, fungal and parasitic infections, cancers, psychiatric conditions such as major depressive disorders, alcohol abuse, gastroenterological disorders such as gastroesophageal reflux disease and peptic ulcers, and cardiovascular conditions such as myocardial infarction and pericarditis.

#### Health-related quality of life scores

The norm-based physical component summary scores (PCSS) and mental component summary scores (MCSS) were computed according to the recommended scoring algorithm for the RAND 36-item health survey 1.0 [18, 19]. PCSS and MCSS, the main explanatory variables, were categorized into terciles with the upper terciles (highest HRQOL) as referent groups. Terciles were established separately for PCSS and MCSS using all available HRQOL scores and we verified an approximately even distribution of the number of participants in each tercile at baseline. Participants could move from one tercile to the other during the period of follow up based on their scores.

#### Covariates

Highly active antiretroviral therapy (HAART) was defined as a combination of at least three full dose antiretroviral agents similar to previous investigations for this cohort [15]. In light of prior reports that use of protease inhibitors (PI) is associated with poorer HRQOL [20–22], HAART was divided into four groups: protease inhibitor-based HAART (PI-HAART), for HAART with at least one protease inhibitor in the HAART regimen; non-protease-inhibitor-based HAART (NPI-HAART), for HAART regimens with no protease inhibitor; HAART-naïve group (HAART-N) for those who had never been on HAART, and Off-HAART/Non-HAART antiretroviral (ART) group, made up of those who were previously treated, but off HAART or who were on non-HAART ART. Other covariates were gender (male/female), age (in increments of 5 years), military rank (officer/warrant officer, enlisted, and civilian/retired), marital status (married, not married), race/ethnicity (Caucasian, African-American, and others), HIV plasma viral load ([pVL], ≤50 copies/mL, > 50 copies/mL), CD4 count (< 200 cells/mm^3^, 200–349 cells/mm^3^, 350–499 cells/mm^3^ and > 499 cells/mm^3^), medical comorbidity (yes/no), mental comorbidity (yes/no), AIDS diagnosis (yes/no), and time since HIV diagnosis (in years). AIDS was defined in accordance with the 1993 Centers for Disease Control and Prevention revised criteria, except for an isolated CD4 cell count < 200 cells/mL, as CD4 was analyzed separately. Medical co-morbidity referred to chronic medical conditions such as diabetes mellitus, hypertension or cancer and was classified as having no comorbidity or having one or more comorbidity. Mental comorbidity was classified similarly. Common mental comorbidities in the NHS were major depressive disorder, generalized anxiety disorder, bipolar disorder and alcohol abuse. Except for gender and race, all other variables were treated as time-varying covariates. Baseline was earliest SF-36 captured according to the above criteria; follow-up continued to hospitalization, loss to follow-up, or Sept 30, 2010, whichever came first. Participants were deemed lost to follow up for this HRQOL analysis if they did not have a completed SF-36 questionnaire for a given year and none thereafter. Participants lost to follow-up were censored at 6 months after the last completed SF-36 or at Sept 30, 2010. All participants aged 18 years and above who were enrolled into the HRQOL sub-study between 2006 and 2010 were eligible for this analysis.

### Statistical analyses

We summarized the characteristics of the participants based on their frequency distribution for categorical variables and the median and interquartile range (IQR) for continuous variables. We plotted the Kaplan-Meier curves to estimate the survivor functions by group using the log-rank test; the Tukey-Kramer adjustment was used to assess between-group differences for variables with more than two categories. Cox regression modeling was used to estimate the hazard of hospitalization for participants while adjusting for covariates. As above, all covariates were time varying, with the exceptions of gender and race. Because separate multivariate models are traditionally used for PCSS and MCSS when these variables are the outcome variables in research settings, we also used them separately as independent variables in two different models while controlling for the same set of covariates (models 1 and 2, respectively). Furthermore, we constructed a third model with both PCSS and MCSS included (model 3). To be eligible for inclusion into the multivariate models, the covariate must achieve a significance level of < 0.2 in the univariate model. Missing data were handled using the last-observation-carried-forward method. In line with the model specifications, we verified the proportional hazard assumptions using both graphical and formal diagnostic tests including covariate-time interaction effects [23–25]. All statistical analyses and graphs were performed using SAS 9.3 [SAS Institute Inc., Cary, NC].

## Results

Out of the 1730 eligible participants at baseline, 13 did not completely answer the HRQOL questionnaire and were excluded. Another 6 participants with missing values for one or more covariates at baseline were also excluded. Of the remaining 1711 participants included in this analysis, 366 (21%) were hospitalized at least once (Table 1). Participants were predominantly male (93%), with equal representation from non-Hispanic Whites and African-Americans (42% each). 17% of participants had a medical comorbidity while 29% had a mental comorbidity, and about 12% had an AIDS diagnosis. Slightly over 5% of the cohort had CD4 count < 200 cells/mm^3^ and over 56% had CD4 count > 499 cells/mm^3^. 35% of participants had pVL > 50 copies/mL. About 34% of patients were on a PI-based HAART while 44% were on a non-PI-based HAART. 14% of participants were HAART naïve at baseline and another 7% were off HAART at baseline. Only 1% of participants (*n* = 18) were on a non-HAART antiretroviral therapy and were combined with the Off-HAART group for the subsequent analyses.

**Table 1:** Demographic and Clinical Characteristics of Participants at Baseline

Categorical Variables	
Characteristics	N (%)
Hospitalized	
Yes	366 (21.39)
No	1345 (78.61)
Gender	
Male	1594 (93.16)
Female	117 (6.84)
Race	
Non-Hispanic White	719 (42.02)
Non-Hispanic African	722 (42.20)
Others	270 (15.78)
Rank	
Officer/Warrant Officer	126 (7.36)
Enlisted	893 (52.19)
Others (Retired/Civilians)	692 (40.44)
Marriage, Yes	556 (32.50)
Medical Comorbidity, Yes	285 (16.66)
Mental Comorbidity, Yes	493 (28.81)
AIDS Diagnosis, Yes	202 (11.81)
HAART	
PI-Based	575 (33.61)
Non-PI-Based	756 (44.18)
HAART-Naïve	241 (14.09)
Off-HAART	121 (7.07)
Non-HAART ART	18 (1.05)
Plasma Viral Load > 50 copies/mL	
Yes	600 (35.07)
No	1111 (64.93)
CD4 Count	
< 200 cells/mm3	92 (5.38)
200–349 cells/mm3	242 (14.14)
350–499 cells/mm3	411 (24.02)
> 499 cells/mm3	966 (56.46)
Continuous Variables	
Characteristics	Median (Interquartile Range)
Age (years)	42.00 (34.00–49.00)
CD4 Count (cells/mm3)	538.00 (389.00–721.00)
Plasma Viral Load (Log10)	1.70 (1.68–2.82)
Time Since HIV Diagnosis (years)	10.00 (4.00–17.00)
Duration of Follow-Up from Baseline (Years, Overall)	2.71 (1.04–3.80)
Hospitalized	1.23 (0.53–2.37)
Not Hospitalized	3.13 (1.53–3.95)
Physical Component Summary Scores (PCSS)	
Lower Tercile	41.78 (35.88–46.13)
Middle Tercile	54.56 (52.78–55.87)
Upper Tercile	58.81 (57.87–59.76)
Mental Component Summary Scores (MCSS)	
Lower Tercile	39.18 (31.99–43.88)
Middle Tercile	50.69 (49.02–51.82)
Upper Tercile	55.22 (54.04–57.29)

**Fig. 1:**
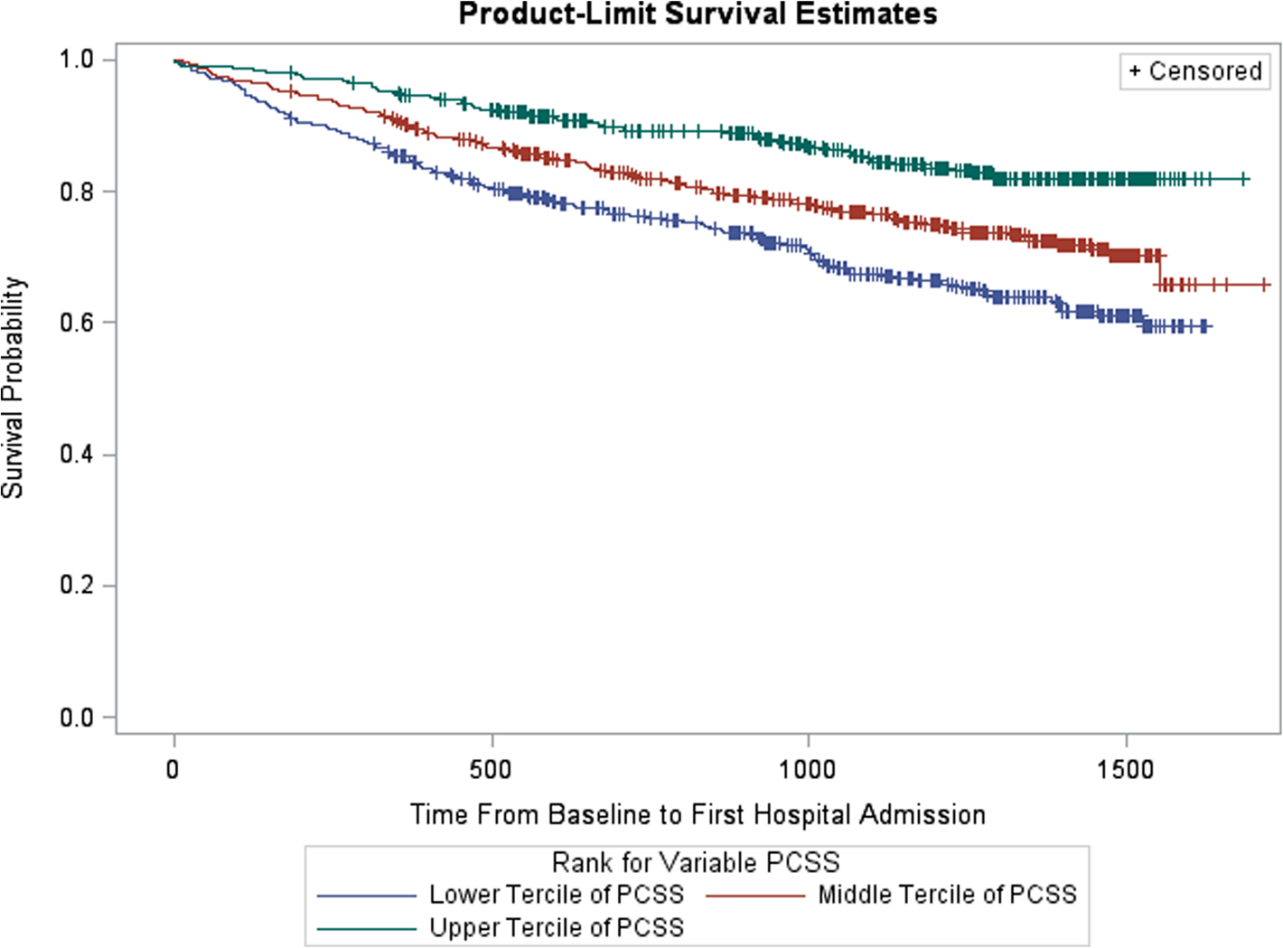
Kaplan-Meier Survival Curve for Physical Component Summary Score (PCSS). Legend:  Lower Tercile of PCSS,  Middle Tercile of PCSS,  Upper Tercile of PCSS

The terciles for the PCSS at baseline had 570, 572 and 569 participants respectively in the lower, middle and upper terciles. The median score of the lower PCSS tercile was 41.78 (IQR, 35.88–46.13) compared to 54.56 (IQR, 52.78–55.87) for the middle tercile and 58.81 (IQR, 57.87–59.76) for the upper tercile. The Kaplan-Meier product-limit survival estimates for hospitalizations among the PCSS terciles showed statistically significant differences among all terciles of PCSS (Fig. 1). In the unadjusted Cox regression model, the hazard ratio (HR) of hospitalization for participants in the lower PCSS tercile was 2.52 times higher compared to participants in the upper PCSS tercile (95% confidence interval [CI] 1.92–3.32; Table 2), while the hazard of hospitalization was 1.74 times higher in the middle tercile compared to the upper tercile (95% CI: 1.31–2.33). After adjustment, the hazards of hospitalization were 2.12 (95% CI: 1.59–2.84) and 1.59 (95% CI: 1.19–2.14) times higher respectively in the lower and middle terciles compared to the upper tercile (Table 3, model 3 [after this, we report model 3, which is the combined PCSS and MCSS model, unless there are obvious differences in the results of the three models]).

**Table 2:** Univariate Cox Regression Model for Hazard of Hospitalization

Variable^a^	Hazard Ratio	95% CI	*P*-Value
Physical Component Summary Score (PCSS)			
Lower Tercile of PCSS	2.52	1.92–3.32	<.0001
Middle Tercile of PCSS	1.74	1.31–2.33	0.0002
Upper Tercile of PCSS	1.0	–	–
Mental Component Summary Score (MCSS)			
Lower Tercile of MCSS	1.79	1.39–2.30	<.0001
Middle Tercile of MCSS	1.27	0.97–1.67	0.08
Upper Tercile of MCSS	1.0	–	–
Age (Years, Increment of 5 Years)	0.98	0.94–1.03	0.5
Gender (Male)	1.06	0.70–1.62	0.8
Marital Status (Married)	1.13	0.91–1.40	0.3
Race/Ethnicity			
Non-Hispanic African American	0.96	0.77–1.19	0.7
Hispanic/Others	0.86	0.63–1.18	0.3
Non-Hispanic Caucasian	1.0	–	–
Rank			
Civilian/Retired	1.47	0.93–2.35	0.1
Enlisted	1.22	0.77–1.95	0.4
Officers	1.0	–	–
CD4 Count			
CD4 Count < 200 cells/mm3	3.89	2.78–5.44	<.0001
CD4 Count 200–349 cells/mm3	2.16	1.63–2.86	<.0001
CD4 Count 350–499 cells/mm3	1.55	1.20–2.00	0.0008
CD4 Count > 499 cells/mm3	1.0	–	–
Plasma Viral Load > 50 Copies/mL	2.36	1.92–2.89	<.0001
Medical Comorbidity	1.05	0.81–1.36	0.7
Mental Comorbidity	1.43	1.16–1.76	0.0009
AIDS Diagnosis	1.67	1.27–2.18	0.0002
Time Since HIV (Per year)	0.98	0.96–0.99	0.002
HAART Treatment			
HAART-Naïve	1.80	1.33–2.43	0.0002
Off-HAART/Non-HAART ART	1.75	1.26–2.44	0.0008
Non-PI Based HAART	0.70	0.55–0.87	0.003
PI Based HAART	1.0	–	–

^a^All variables are time-varying except for race and gender

**Table 3:** Multivariate Cox Regression Model for Hazard of Hospitalization for Terciles of PCSS and MCSS

Variable^a^	Model 1: PCSS Model		Model 2: MCSS Model		Model 3: Combined PCSS and MCSS Model	
	HR	95% CI	HR	95% CI	HR	95% CI
PCSS						
Lower Tercile	2.18	1.64–2.90			2.12	1.59–2.84
Middle Tercile	1.62	1.21–2.17			1.59	1.19–2.14
Upper Tercile	1.0	–			1.0	–
MCSS						
Lower Tercile			1.44	1.10–1.87	1.33	1.02–1.73
Middle Tercile			1.14	0.87–1.49	1.20	0.91–1.57
Upper Tercile			1.0	–	1.0	–
CD4 Count						
< 200 cells/mm3	2.84	1.96–4.12	2.99	2.06–4.33	2.84	1.96–4.12
200–349 cells/mm3	1.69	1.25–2.27	1.67	1.24–2.25	1.67	1.24–2.26
350–499 cells/mm3	1.41	1.09–1.82	1.37	1.05–1.77	1.41	1.09–1.83
> 499 cells/mm3	1.0	–	1.0	–	1.0	–
Plasma Viral Load > 50 Copies/mL	1.83	1.47–2.28	1.88	1.51–2.34	1.82	1.46–2.26
Mental Comorbidity	1.31	1.04–1.63	1.30	1.04–1.64	1.23	0.98–1.55
AIDS Diagnosis	1.35	1.00–1.83	1.47	1.09–1.99	1.34	0.99–1.81
Rank						
Civilian/Retired	2.06	1.26–3.37	2.16	1.32–3.52	2.04	1.25–3.34
Enlisted	1.18	0.74–1.88	1.17	0.73–1.86	1.19	0.74–1.89
Officer	1.0	–	1.0	–	–	–
Time Since HIV (Per year)	0.94	0.92–0.96	0.94	0.93–0.96	0.94	0.93–0.96

^a^All variables are time-varying

**Fig. 2:**
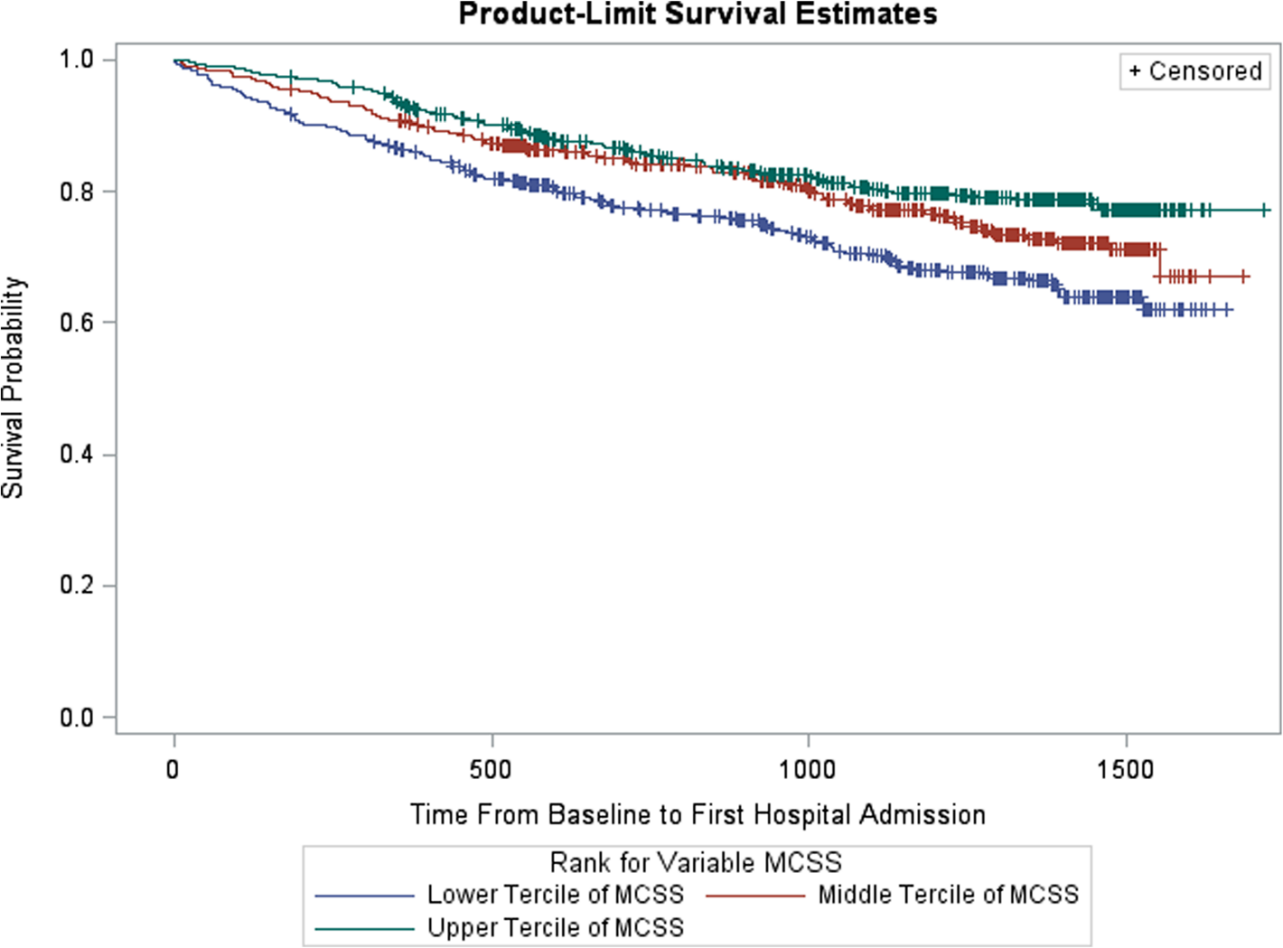
Kaplan-Meier Survival Curve for Mental Component Summary Score (MCSS). Legend:  Lower Tercile of MCSS,  Middle Tercile of MCSS,  Upper Tercile of MCSS

The terciles for the MCSS at baseline had 569, 571, and 571 participants respectively for the lower, middle and upper terciles. The median score of the lower MCSS tercile was 39.18 (IQR, 31.99–43.88) compared to 50.69 (IQR, 49.02–51.82) for the middle tercile and 55.22 (IQR, 54.04–57.29) for the upper tercile. The Kaplan-Meier product-limit survival estimates for hospitalizations among the MCSS terciles showed statistically significant differences between the lower tercile and other two MCSS terciles, but not between the middle and upper terciles (Fig. 2). In the unadjusted Cox regression model (Table 2), participants in the lower MCSS tercile were 79% at increased hazard of being hospitalized compared to those in the upper MCSS tercile (HR: 1.79; 95% CI: 1.39–2.30). After adjustment (model 3), the hazard of hospitalization in the lower tercile was 33% higher than that of the upper tercile (HR: 1.33; 95% CI 1.02–1.73).

The first-hospitalization rate among the 1711 participants was 8.7 per 100 person-years (PYs). As previously noted, the number of participants at baseline in the lower, middle and upper PCSS terciles were 570, 572 and 569 and the total duration of follow up in years were respectively 1312.34, 1414.88 and 1471.44. There were 168 hospitalizations in lower terciles for the period of follow up, 124 in the middle tercile and 74 in the upper tercile, therefore making the rates of hospitalization 12.8, 8.8 and 5.0 per 100 PYs for the lower, middle and upper terciles respectively (Table 4). Similarly, the rates of hospitalization were 11.8, 8.3 and 6.5 per 100 PYs for the lower, middle and upper terciles of MCSS respectively (Table 4).

**Table 4:** Overall Rates of Hospitalization in the NHS and by Terciles of PCSS and MCSS

HRQOL	Terciles of HRQOL	Total Participants at Baseline^a^	Total No. Hospitalized during Follow Up	Duration of Follow Up (Years)	Rate of Hospitalization Per 100 Person - Years
PCSS and MCSS	Overall	1711	366	4201.67	8.71
PCSS	Lower	570	168	1312.34	12.80
	Middle	572	124	1414.88	8.76
	Upper	569	74	1474.44	5.02
MCSS	Lower	569	152	1293.77	11.75
	Middle	571	116	1397.09	8.30
	Upper	571	98	1510.8	6.49

^a^Participants could move from one tercile to the other during the period of follow up based on their scores

The HRs of hospitalization were significantly increased in participants with CD4 count < 200 cells/mm^3^, 200–349 cell/mm^3^ and 350–499 cell/mm^3^ by 2.84 (95% CI: 1.96–4.12), 1.67 (95% CI: 1.24–2.26), and 1.41 (95% CI: 1.09–1.83) times respectively when compared to those with CD4 count > 499 cells/mm^3^. Having pVL > 50 copies/mL was significantly associated with 82% increase in the hazard of hospitalization (HR: 1.82; 95% CI: 1.46–2.26) while being retired/civilian was significantly associated with over a 100% increase in hazard of hospitalization (HR: 2.04; 95% CI: 1.25–3.34) (Table 3). Every year increment in time from HIV diagnosis led to a 5.6% reduced hazard of hospitalization (HR: 0.94; 95% CI: 0.93–0.96). Factors that were not significantly associated with hospitalization in the combined model but significant in the PCSS and/or MCSS models (Table 3) were mental comorbidity (both PCSS and MCSS models) and AIDS diagnosis (MCSS model only). HAART, on the other hand, was only significant in the univariate model but not in any of the multivariate models and therefore not shown in our most parsimonious models.

## Discussion

Our study shows that both PCSS and MCSS were independently predictive of hospitalization among participants in the NHS cohort. To the best of our knowledge, this is the first study to use the SF-36 to evaluate whether HRQOL predicts hospitalization in an HIV-infected population, and perhaps the second study for any HRQOL instrument [5]. Our study therefore adds to the growing literature on the role of HRQOL in predicting hospitalization in chronic diseases [11, 26–28]. In our current study, those in the middle PCSS tercile were at 59% greater hazard of hospitalization compared to the upper PCSS tercile while those in the lower PCSS tercile were at 112% greater hazard of hospitalization compared to the upper PCSS tercile. Participants in the lower MCSS tercile had a 33% increased hazard of hospitalization compared to the upper MCSS tercile. These findings support the use of HRQOL in risk assessment for hospitalization in clinical and research settings. Although clinical factors such as mental comorbidity and AIDS diagnosis were not significantly associated with hospitalization in the combined model (model 3, Table 3), mental comorbidity was significant in the separate PCSS and MCSS models while AIDS diagnosis was significant in the MCSS model and was nearly significant in the PCSS model. These covariates should therefore be included in studies examining clinical interventions directed at reducing the risk of hospitalization in PLWHA considering that they contribute to both the independent and outcome variables.

The overall rate of hospitalization of 8.7 per 100 PYs in our current study is much lower than that reported earlier for the NHS by Crum-Cianflone et al. [13], which reported a hospitalization rate of 137 per 1000 PYs or 13.7 per 100 PYs. This difference may be partly explained by the fact that we only considered the first hospitalization per individual as against all hospitalizations used in the study by Crum-Cianflone et al. Indeed, in that study 47% of all hospitalizations were repeat inpatient admissions. Furthermore, the general decline in hospitalization rates in HIV-infected populations over the years may partly explain the differences in hospitalization rates noted in both studies. The rates of hospitalizations were also markedly different by terciles of PCSS and MCSS. These rates were 5.0, 8.8 and 12.8 per 100 PYs for the upper, middle and lower PCSS terciles respectively, and 6.5, 8.3 and 11.7 per 100 PYs for the upper, middle and lower terciles of MCSS respectively. This clear pattern of less hospitalization with better physical and mental functional health status suggests that applying innovative research to delineate interventions that will improve HRQOL may be strategically significant in reducing hospitalization in our population and likely more broadly among PLWHA.

Our findings on the association among CD4 count and hospitalization were similar to and reinforced prior work. Compared to CD4 count > 499 cells/mm^3^, CD4 count < 200 cells/mm^3^, CD4 count 200–349 cells/mm^3^, and CD4 count 350–499 cells/mm^3^ were respectively associated with increased hazard of hospitalization by 184, 67 and 41%. Somewhat similar to our findings, Crum-Cianflone et al. [13], in an earlier work on this cohort, had found that CD4 count > 499 cells/mm^3^ reduced the hazard of hospitalization when compared to CD4 < 350 cells/mm^3^ but not 350–499 cells/mm^3^. The reason for the difference with our current study may be related to differences in the time frame studied (2006–2010 [current study] versus 1999–2007 [previous study]) or in study design (first hospitalization [current study] versus all hospitalizations [previous study]) [13]. Other investigators have also shown that lower CD4 count is associated with hospitalization, especially when CD4 count falls below 200 [29–34]. HIV pVL > 50 copies/mL was also associated with hospitalization in our cohort. Although the levels of dichotomization differed, Fielden et al. [34] also found that higher pVL was associated with hospitalization while Mocroft et al. [29] demonstrated that in the last of three time points in their study, there was an increased odds of hospitalization for every log unit increase in pVL.

Interestingly, a longer time since HIV diagnosis was predictive of a reduced hazard of hospitalization. One plausible explanation for this finding may be that individuals with longer disease duration may be more experienced with dealing with symptoms (including subtle ones) associated with their infection and therefore more likely to seek medical attention earlier before admission is warranted. Survival bias may also play a role as those who live longer due to their innate ability to cope with the disease are less likely to be hospitalized because of their relatively healthier state. The finding that being civilian/retired was associated with over 100% increased hazard of hospitalization is not totally unexpected as this group is older, and age has been associated with higher inpatient admissions [35]. However, the fact that age was not independently associated with hospitalization in our current study may partly reflect the relatively young age of our cohort (median age 42 years; IQR 34 to 49 years) but may also be suggestive of the fact that other factors associated with non-active duty status, such as health related behaviors, played a more important role in this respect. Although being civilian/retired has been shown to be associated with lower PCSS in our cohort [36], its association with hospitalization in the current study is independent of PCSS (Tables 3 and 5). Finally, while HAART was significant in the unadjusted model, it was no longer significant after adjustment showing that its effect may have been captured by other variables.

**Table 5:** Multivariate Cox Regression Model for Hazard of Hospitalization (PCSS and MCSS Numeric)

Variable^a^	Model 4: PCSS Model		Model 5: MCSS Model		Model 6: Combined PCSS and MCSS Model	
	HR	95% CI	HR	95% CI	HR	95% CI
PCSS, 5 Unit Increments	0.87	0.83–0.92			0.88	0.84–0.93
MCSS, 5 Unit Increments			0.91	0.87–0.96	0.94	0.89–0.99
CD4 Count						
< 200 cells/mm3	2.76	1.90–4.01	2.96	2.04–4.29	2.73	1.88–3.97
200–349 cells/mm3	1.66	1.23–2.23	1.67	1.24–2.25	1.65	1.22–2.23
350–499 cells/mm3	1.38	1.07–1.80	1.37	1.05–1.77	1.38	1.07–1.79
> 499 cells/mm3	1.0	–	1.0	–	1.0	–
Plasma Viral Load > 50 Copies/mL	1.86	1.49–2.32	1.89	1.52–2.36	1.86	1.49–2.31
Mental Comorbidity	1.31	1.05–1.64	1.28	1.02–1.61	1.23	0.97–1.54
AIDS Diagnosis	1.34	0.99–1.82	1.48	1.09–2.00	1.34	0.99–1.82
Rank						
Civilian/Retired	2.21	1.35–3.61	2.14	1.31–3.49	2.15	1.32–3.52
Enlisted	1.28	0.80–2.04	1.16	0.73–1.85	1.27	0.79–2.02
Officer	1.0	–	1.0	–	–	–
Time Since HIV (Per year)	0.94	0.92–0.96	0.95	0.93–0.96	0.94	0.93–0.96

^a^All variables are time-varying

Potential limitations of our study include the predominantly male patients in the NHS cohort which may limit its generalizability to females. The regimented lifestyle of our military population and the requirements of physical and mental fitness may also mean that they may promptly seek medical attention, a behavior that may not necessarily be seen in the general population. We may not have captured hospital admissions outside the military settings; however, with the use of trained coordinators to conduct participants’ interviews, the number of missed admissions outside the single payer electronic health records systems of the U.S. military is expected to be small. Because we administratively censored participants after September 2010, follow-up was limited for those subjects surveyed in 2010; however, as subjects were censored 6 months after their last survey, this affected only those enrolled in 2010 (*n* = 99) [36]. In sensitivity analysis we excluded these 99 participants and our results remained essentially the same. In this current study, we did not specifically evaluate whether within-person difference in HRQOL score may predict hospitalization. While such difference in score may be more meaningful to the clinician interested in individual risk of inpatient admission over time, we believe that knowing the actual scores that predict hospitalization is a useful starting point. More so, within-person difference in score may be dependent on the baseline HRQOL score. Finally, our findings that PCSS and MCSS were predictive of hospitalization should not be interpreted as implying causation but instead that physical and mental functional health may be surrogates for the actual causes of hospitalization. Yet, the fact that these measures were statistically significant in predicting hospitalization in all multivariate models, the magnitude of the effect sizes, and the dose response relationships support their utility in clinical practice.

Our study had major advantages. There was clearly an established temporal relationship between the HRQOL scores and hospitalization. Because hospitalization may also negatively impact HRQOL [37], we further conducted sensitivity analyses to rule out reverse causation by excluding participants whose scores were taken within 1 week hospitalization but our findings remained essentially the same. This temporal pattern was also true for the other time-dependent covariates included in the study. The clear dose-response relationship between PCSS and hospitalization in our study strongly supports its utility as a predictive tool. To further demonstrate the discriminatory quality of PCSS we used it as a continuous variable, and it showed that for every 5-unit increase in PCSS the hazard of hospitalization was reduced by 12% (Table 5). MCSS, on the other hand, reduced the hazard of hospitalization by 6% for every 5-unit increase in MCSS. Our study therefore adds to the predictive utility of the HRQOL measures in HIV-infected persons as assessed by the SF-36. Other important strengths of our study include its large sample size, open access to healthcare and medications, racial diversity, and the heterogeneity of the cohort with regards to the range of values for HIV disease indicators (CD4 count and pVL), and other clinical parameters, such as medical and mental comorbidities with respective prevalence of 17 and 29% of the cohort at baseline. The heterogeneity of the cohort (Table 1) is further reflected by the wide range of PCSS (16.7 to 70.7) and MCSS (8.8 to 67.8), a finding that is rare in other predictive studies using the SF-36 that tend to be with persons with relatively very low scores [3, 4, 6, 28, 38]. Finally, HRQOL summary scores may be useful in prognostic studies in HIV-infected populations because they capture information beyond HIV disease-specific indicators, such as CD4 count and pVL. The causes of hospitalization in HIV-infected individuals are now beyond disease-related factors but include both medical and surgical conditions, especially in the HAART era [13]. HRQOL is also reflective of perceptions that may potentially affect subsequent health-seeking behaviors and utilization of healthcare resources including preventive services [4, 39].

## Conclusion

After controlling for factors associated with hospitalization among those with HIV, both PCSS and MCSS were predictive of all-cause hospitalization in the NHS cohort with similar effect sizes for PCSS and low CD4. Applying innovative strategies to improve modifiable risk factors that influence the physical and mental functional status of HIV-infected individuals has the potential to reduce the rate of hospitalization in this patient population. We, therefore, recommend the use of HRQOL in stratifying hospitalization risk among HIV-infected individuals. Finally, future research to evaluate whether within-person difference in HRQOL scores is predictive of hospitalization is needed, as this may even increase the utility of HRQOL measures in clinical settings.

## Abbreviations

HAART: Highly Active Antiretroviral Therapy
HIV: Human Immunodeficiency Virus
HRQOL: Health-Related Quality of Life
MCSS: Mental Component Summary Scores
NPI-HAART: Non-Protease Inhibitor HAART
PCSS: Physical Component Summary Scores
PI-HAART: Protease Inhibitor HAART
pVL: Plasma Viral Load
SF-36: Short Form 36
